# Synaptonemal complex protein 3 is associated with lymphangiogenesis in non-small cell lung cancer patients with lymph node metastasis

**DOI:** 10.1186/s12967-017-1241-5

**Published:** 2017-06-17

**Authors:** Haruhisa Kitano, Joon-Yong Chung, Kyung Hee Noh, Young-Ho Lee, Tae Woo Kim, Seok Hyung Lee, Soo-Heang Eo, Hyung Jun Cho, Chel Hun Choi, Shuhei Inoue, Jun Hanaoka, Junya Fukuoka, Stephen M. Hewitt

**Affiliations:** 1 Experimental Pathology Laboratory, Laboratory of Pathology, Center for Cancer Research, National Cancer Institute, National Institutes of Health, Bethesda, MD 20892 USA; 2 Department of Biomedical Sciences, Graduate School of Medicine, Korea University, Seoul, 136-701 Korea; 3 Department of Biochemistry & Molecular Biology, Korea University College of Medicine, Seoul, 136-701 Korea; 4 Department of Statistics, Korea University, Seoul, 136-701 Korea; 5 Department of Obstetrics and Gynecology, Samsung Medical Center, Sungkyunkwan University School of Medicine, Seoul, 135-710 Korea; 6 Department of Thoracic Surgery, National Hospital Organization Higashi-Ohmi General Medical Center, Higashi-Oumi, 527-8505 Japan; 7 Department of Thoracic Surgery, Shiga University of Medical Science, Otsu, 520-2192 Japan; 8 Department of Pathology, Nagasaki University Graduate School of Biomedical Sciences, 1-12-4, Sakamoto, Nagasaki, 852-8523 Japan

**Keywords:** SCP3, Vascular endothelial growth factor, Lymphangiogenesis, Lymph node metastasis, Non-small cell lung cancer

## Abstract

**Background:**

The interaction of vascular endothelial growth factor-C (VEGF-C)/VEGF-D/VEGF receptor-3 is considered to be a major driver of lymphangiogenesis, however the mechanism of this process remains unclear. We aimed to investigate the possible lymphangiogenic significance of synaptonemal complex protein 3 (SCP3) in non-small cell lung cancer (NSCLC).

**Methods:**

The expression of SCP3, VEGF-C, and VEGF-D were measured and examined a correlation between SCP3 and VEGF-C or VEGF-D in various human lung cancer cell lines. Subsequently, we assessed SCP3, VEGF-A, VEGF-B, VEGF-C, and VEGF-D expression in archival tumor tissues from 89 NSCLC patients with lymph node (LN) metastasis by combined immunohistochemistry with quantitative digital image analysis.

**Results:**

Positive correlations between SCP3 and VEGF-C expression (*R* ^*2*^ = 0.743) and VEGF-D expression (*R* ^*2*^ = 0.932) were detected in various human lung cancer cell lines. The high expression of SCP3, VEGF-A, VEGF-B, VEGF-C, and VEGF-D were detected in 24 (27.0%), 22 (24.7%), 27 (30.3%), 27 (30.3%), and 24 cases (27.0%), respectively. Notably, SCP3 positively correlated with VEGF-C and VEGF-D expression (for both, *P* < 0.001) and negatively correlated with VEGF-A and VEGF-B expression (*P* = 0.029 and *P* = 0.026, respectively). In multivariate analysis of patients with LN metastasis, SCP3 expression predicted worse overall survival (hazard ratio = 1.86, *P* = 0.008).

**Conclusions:**

SCP3 is associated with lymphangiogenesis and provides insight into the SCP3-VEGF-C/VEGF-D axis based cancer therapy strategy.

**Electronic supplementary material:**

The online version of this article (doi:10.1186/s12967-017-1241-5) contains supplementary material, which is available to authorized users.

## Background

Lung cancer is the leading cause of cancer-related deaths in the United States, accounting for 157,000 deaths annually [1]. Only 15% of lung cancer cases are diagnosed at an early stage, whereas 22% are diagnosed by the time the tumor has already spread to the lymph nodes. Median survival time is 84 months for lymph node (LN) negative patients, compared to 30 months for N1, 33 months for N2 single positive, and 11 months for N2 multi [2]. In addition, metastasis is common in lung cancer and is considered to be a critical factor in determining prognosis. Correctively, determining the extent of LN involvement is assessing the patients’ prognosis and choosing the optimal therapeutic strategy.

Angiogenesis and lymphangiogenesis play key roles in tumor growth and metastasis in non-small cell lung cancer (NSCLC). The balance between angiogenic and lymphangiogenic factors regulates tumor progression and metastasis [3]. Angiogenesis in NSCLC is, commonly associated with concomitant lymphangiogenesis. However, lymphangiogenesis is considered to be the key initial step in lymphatic and regional LN metastasis [4]. Although the significance of angiogenesis for tumor growth and metastasis has been well understood, the molecular mechanisms of lymphangiogenesis and lymphatic metastasis in tumor metastasis remain unclear [5]. The members of the vascular endothelial growth factor (VEGF) family are considered to be major mediators of both tumor angiogenesis and lymphangiogenesis [6]. Among the VEGF family members, VEGF-C and VEGF-D are known as key molecules that mediate the formation of tumor lymphatics as well as metastatic spread of tumor cells to lymph nodes via vascular endothelial growth factor-3 (VEGFR-3) [4, 5, 7]. The extent to which LN metastases promote the spreading of the tumor cells is no yet clear [8]. Thus, the prognostic significance of potential lymphangiogenic markers remains controversial [9].

Synaptonemal complex protein 3 (SCP3) is a DNA-binding protein and a structural component of the synaptonemal complex, which mediates the synapsis or homologous pairing of chromosomes during human spermatogenesis. Although SCP3 expression is highly restricted in the nucleus of meiotic germ cells, ectopic SCP3 expression is frequently observed in human cancers, such as acute lymphoblastic leukemia and cervical cancer [10, 11]. We previously demonstrated that SCP3 was significantly associated with cervical cancer progression and LN metastasis of cervical cancer [12], while SCP3 has been defined as a negative prognostic factor in the early stage of NSCLC by immunohistochemistry (IHC) and manual visual scoring [13]. This discrepancy may be explained by the subjectivity of assessing SCP3 positivity. Although visual scoring is the golden standard method for quantifying IHC staining, there are limitations such as quasi-continuous variable data, subjectivity by examiner, and less than optimal reproducibility [14–16]. On the other hand, quantitative digital image analysis may overcome many of these limitations. Thus, we tried to reduce artifacts by using a quantitative digital image analysis program for IHC scoring in the present study. In an effort to understand the role SCP3 plays in NSCLC, we examined its potential relationship to LN metastasis by IHC and quantitative image analysis. Based on this pattern of expression, and in combination with investigation into the expression of VEGF-family members in NSCLC, we further investigated these relationships.

## Methods

### Cells

H146, H460, H1299, H1666, H2228, H358, and H3122 cell lines were obtained from American type culture collection (ATCC, Manassas, VA). All cells were maintained in RPMI-1640 supplemented with 10% FBS in the presence of 5% CO_2_ at 37 °C in a humidified incubator. H358/no insert, H358/SCP3, H1666/no insert and H1666/SCP3 cells were generated by retroviral transduction with pMSCV/empty (Clontech, Mountain View, CA) or pMSCV/SCP3 as previously described [11].

### Synthetic small interfering RNAs constructs

Synthetic small interfering RNAs (siRNAs) specific for green fluorescent protein (GFP) or human SCP3 (hSCP3) were purchased from the Invitrogen (Invitrogen, Carlsbad, CA); GFP, 5′-GCAUCAAGGUGAACUUCAA-3′ (sense), 5′-UUGAAGUUCACCUUGAUGC-3′ (anti-sense); hSCP3, 5′-GGAGAAGAAUCAUGAUAAU-3′ (sense), 5′-AUUAUCAUGAUUCUUC-UCC-3′ (anti-sense). For in vitro delivery, tumor cells on a six-well vessel were transfected with 300 pmol of synthesized siRNAs using Lipofectamine 2000 (Invitrogen) according to the manufacturer’s instructions.

### Western blot analysis

To determine the expressional levels of proteins, 50 μg of protein were separated on SDS-PAGE and transferred to nitrocellulose membrane. The membranes were blocked with 5% nonfat dry milk in TBST (50 mM Tris, pH 7.5, 150 mM NaCl, 0.05% Tween-20) for 1 h, washed, and subsequently incubated overnight at 4 °C in TBST with 5% BSA containing the following antibodies: mouse monoclonal anti-SCP3 (clone# 25/SCP3; 0.5 µg/ml; BD Bioscience, San Jose, CA), goat polyclonal anti-VEGF-C (cat.# AF752; 0.5 µg/ml; R&D Systems, Minneapolis, MN), goat polyclonal anti-VEGF-D (cat.# AF286; 0.5 µg/ml; R&D Systems) and mouse monoclonal anti-β-actin (clone# AC-74; 0.5 µg/ml; Sigma-Aldrich, St. Louis, MO). Specific molecules were detected with HRP-labeled anti-mouse or anti-rabbit secondary antibodies (Pierce, Rockford, IL) and enhanced with ECL kit (Elpis Biotech, Korea). Densitometry was performed using an image analyzer Fujifilm LAS-400 (Fuji, Tokyo, Japan) and Image J densitometry software (Version 1.6, National Institutes of Health, Bethesda, MD) was used for analysis.

### Tissue samples

A total of 89 NSCLC cases with LN metastasis were prospectively selected from patients who were enrolled at Toyama University Hospital and National Hospital Organization Higashi-Ohmi General Medical Center. All patients underwent complete resections between 1988 and 2010, but did not receive neoadjuvant chemo or radio-therapy. Survival time and outcome data were available for all patients. Tumors were staged according to the International Union against cancer’s tumor-node-metastasis (TNM) classification. The histology was classified and graded according to 2004 World Health Organization guidelines [17]. The follow-up period ranged from 10 to 153 months.

### Tissue microarray and immunohistochemistry

Tissue microarray (TMA) was constructed from archival formalin-fixed, paraffin-embedded tissue blocks. Three 1.0 mm diameter tissue cores were arrayed on a recipient paraffin block using a tissue arrayer (Pathology Devices, Westminster, MD). Briefly, a representative tumor area was carefully selected for each tumor from hematoxylin and eosin (H&E) stained section of a donor block. TMA blocks were cut into serial 5-µm-thick sections, and then sections were deparaffinized in xylene and were rehydrated through a graded alcohol series to distilled water. Immunohistochemistry for SCP3 was performed as previously described [13]. For VEGF-A, VEGF-B, VEGF-C and VEGF-D, endogenous peroxidase was blocked using a 3% solution of aqueous hydrogen peroxide. Immunohistochemical staining was performed with the following primary antibodies: mouse monoclonal anti-VEGF-A (clone# 16F1; diluted 1:100; Thermo scientific, Waltham, MA), mouse monoclonal anti-VEGF-B (clone# MM0008-7B43; diluted 1:100; Abcam, Cambridge, MA), goat polyclonal anti-VEGF-C (Cat.# AF752; diluted 1:100; R&D Systems), and goat polyclonal anti VEGF-D (cat.# AF286; diluted 1:100; R&D systems). Antigen retrieval for VEGF-C and VEGF-D was performed using a pressure chamber (Pascal; Dako, Carpinteria, CA) with pH 6 target retrieval solution (Dako). For VEGF-A and VEGF-B antibodies, the TMA slide was pretreated with pH 9 target retrieval solution (Dako). After antigen retrieval, slides were incubated with primary antibodies for 30 min (VEGF-A) or overnight (VEGF-B, VEGF-C and VEGF-D) at 4 °C. Signals were detected with LSAB detection system (Dako) for VEGF-C and VEGF-D, and with Envision^+^ detect system (Dako) for the other antibodies. The stain was visualized using DAB (Dako) and then was lightly counterstained with hematoxylin, dehydrated in ethanol, and cleared in xylene. Negative controls were processed by omitting the primary antibodies, and TMA included testis positive control tissues. The cut-off values of histoscore were identified considering the distribution and prognostic significance of the values (Additional file 1: Figure S1).

### Digital image analysis

The immunohistochemical staining was assessed using computer-assisted image analysis software, as described previously [18]. In brief, immunohistochemically stained slides were scanned by the NanoZoomer 2.0 HT (Hamamatsu Photonics K.K., Japan) at ×20 objective magnification (0.5 μm resolution). Captured images were analyzed using Visiopharm Digital Image Analysis (DIA) software (for Windows 7, version 4.5.1.324; Visiopharm, Hørsholm, Denmark). First, transformation of an image from one form to another (image processing) is carried out to enhance relative image structures for subsequent image segmentation. After training the system by digitally “painting” examples of the nucleus in the image, tumor nuclei were defined during segmentation. The cytoplasm was further defined by outlining the defined nucleus. The pixels that contribute to positive staining were identified based on a DAB color deconvolution. The algorithm evaluated each pixel on the value of 0–255. We used the mean of the pixel intensity values as the quantity of protein expression. Cut-off values of histoscore were identified considering the distribution and prognostic significance of the values (Additional file 1: Figure S1).

### Statistical analysis

Statistical analysis was performed using JMP Statistical Discovery Software, Version 7.0.1 (SAS Institute, Cary, NC). Chi square tests were used to evaluate the association between SCP3 and VEGFs. Survival analysis was performed for all cases. Kaplan–Meier survival analysis was used to determine the univariate relationship of SCP3 expression and overall survival, and survival curves were compared by using the log-rank test. Multivariate proportional Cox models with adjustments for antibody expression, age at diagnosis, gender, T factor, and cancer type were used. *P* values were considered significant when they were less than 0.05. Regression analyses were performed using the mean of the pixel intensity values of SCP3 and VEGFs. The response surfaces showed the correlation between SCP3, VEGFs and N factor (N1 or N2–N3).

## Results

### Relationship between SCP3 and VEGF-C or VEGF-D expression in human lung cancer cells

**Fig. 1:**
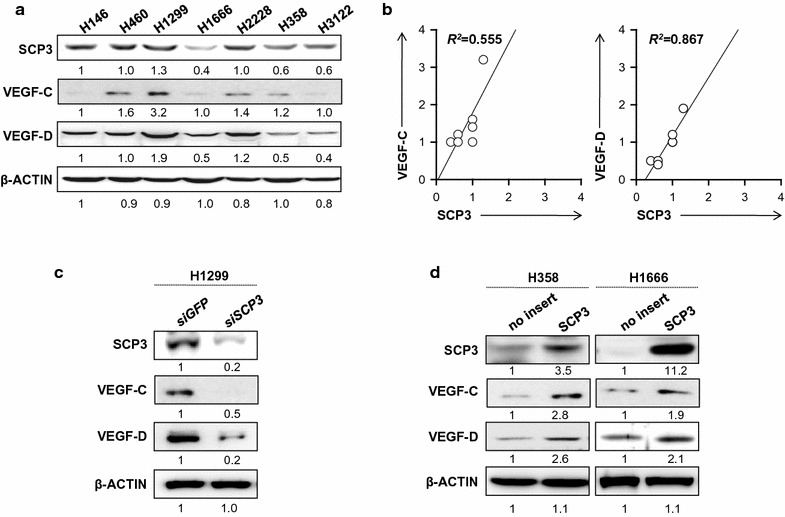
Correlation between SCP3 and VEGF-C or VEGF-D expressions in human lung cancer cells. **a** Western blot analysis to characterize the expression of SCP3, VEGF-C and VEGF-D in various human lung adenocarcinoma cells; H146, H460, H1299, H1666, H2228, H358, and H3122. **b** A plot graph demonstrating the linear relationship between expressing SCP3 (x-axis) and VEGF-C or VEGF-D (y-axis). Western blot analysis of SCP3 expression in lung cancer cell lines cells retrovirally transduced with a pMSCV vector encoding SCP3. **c** *siGFP*- or *siScp3* transfected with H1299 **d** H358 and H1666 cells, retrovirally transduced with PMSCV vector encoding either no insert (H358/no insert, H1666/no insert) or *Scp3* (H358/SCP3, H1666/SCP3), were incubated in 0.1% FBS-containing DMEM medium for 24 h

Given that SCP3 had been implicated in cervical cancer and metastasis to lymph nodes, we decided to examine its role in lung cancer. It has been known that VEGF-C and VEGF-D are associated with tumor lymphangiogenesis and metastasis [19, 20]. Thus, we measured the expression of SCP3, VEGF-C, and VEGF-D using human lung cancer cell lines. We observed a correlation between SCP3 and VEGF-C or VEGF-D in various human lung cancer cell lines (Fig. 1). Notably, linear regression analysis demonstrated that the level of SCP3 expression positively correlated with VEGF-C (*R* ^2^ = 0.555) or VEGF-D (*R* ^2^ = 0.867) in various human lung cancer cell lines (Fig. 1b). We further examined whether the relationship between SCP3 and VEGF-C or SCP3 and VEGF-D was conserved in human lung cancer cells. Consistently, we observed decreased levels of VEGF-C and VEGF-D when endogenous SCP3 high tumor cells (H1299) were treated with siRNA targeting to SCP3 (siSCP3) but not with non-specific control siRNA targeting GFP (siGFP). Conversely, increased VEGF-C and VEGF-D levels among H358 and H1666 cells expressing low levels of SCP3 were observed after over-expressing hSCP3 by a retroviral system (Fig. 1c, d).

### Expression of SCP3 and VEGFs in NSCLC

**Fig. 2:**
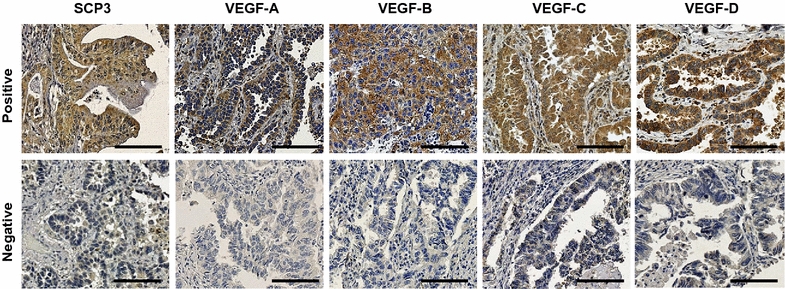
Representative immunohistochemical images of SCP3, VEGF-A, VEGF-B, VEGF-C and VEGF-D in formalin-fixed, paraffin-embedded non-small cell lung cancer (NSCLC) tissues. *Scale bar* = 100 μm

We performed immunohistochemistry on 89 formalin-fixed paraffin-embedded tissues from NSCLC patients with LN metastasis for investigated the expression of SCP3, VEGF-A, VEGF-B, VEGF-C, and VEGF-D. Forty-six (51.7%) patients were N1 and 43 (48.3%) patients were N2–3 (Additional file 2: Table S1). Expression of SCP3 and all examined VEGFs was primarily detected in cytoplasm (Fig. 2), and the rate of high expression ranged from 24.7 to 30.3% (Table 1). No correlations between clinicopathological factors and SCP3 or VEGFs expression was observed, except for the association between VEGF-D and tumor type (*P* = 0.02, Table 1).

**Table 1:** Clinicopathological parameters of patients with SCP3, VEGF-A, VEGF-B, VEGF-C, and VEGF-D expression

Category	No. of case	Expression														
		SCP3			VEGF-A			VEGF-B			VEGF-C			VEGF-D		
		Low, *n* (%)	High, *n* (%)	*P*	Low, *n* (%)	High, *n* (%)	*P*	Low, *n* (%)	High, *n* (%)	*P*	Low, *n* (%)	High, *n* (%)	*P*	Low, *n* (%)	High, *n* (%)	*P*
Total	89	65 (73)	24 (27)		67 (75)	22 (25)		62 (70)	27 (30)		62 (70)	27 (30)		65 (73)	24 (27)	
Gender				0.24			0.51			0.83			0.76			0.24
Male	64	49 (76)	15 (24)		47 (73)	17 (27)		45 (70)	19 (30)		44 (69)	20 (31)		49 (77)	15 (23)	
Female	25	16 (64)	9 (36)		20 (80)	5 (20)		17 (68)	8 (32)		18 (72)	7 (28)		16 (64)	9 (36)	
Age (year)				0.69			0.13			0.76			0.40			0.89
<60	25	19 (76)	6 (24)		16 (64)	9 (36)		44 (69)	20 (31)		19 (76)	6 (24)		18 (72)	7 (28)	
≥60	64	46 (72)	18 (28)		51 (80)	13 (20)		18 (72)	7 (28)		43 (67)	21 (33)		47 (73)	17 (27)	
Smoking				0.31			0.30			0.77			0.14			0.58
Never	19	20 (100)	0 (0)		11 (58)	6 (42)		11 (58)	8 (42)		17 (89)	2 (11)		16 (84)	3 (16)	
Ever	29	28 (97)	1 (3)		21 (72)	8 (28)		18 (62)	11 (38)		21 (72)	8 (28)		26 (90)	3 (10)	
Stage				0.18			0.66			0.60			0.68			0.56
II	40	32 (80)	8 (20)		31 (78)	9 (22)		29 (73)	11 (28)		27 (68)	13 (32)		28 (70)	12 (30)	
III–IV	49	33 (67)	16 (33)		46 (73)	13 (27)		33 (67)	16 (33)		35 (71)	14 (29)		37 (76)	12 (24)	
T stage				0.49			0.64			0.32			0.78			0.22
T1	15	12 (80)	3 (20)		12 (80)	3 (20)		12 (80)	3 (20)		10 (67)	5 (33)		9 (60)	6 (40)	
T2–4	74	53 (72)	21 (28)		55 (74)	19 (26)		50 (68)	24 (32)		52 (70)	22 (30)		56 (76)	18 (24)	
Tumor type				0.18			0.95			0.33			0.16			0.02
AD	53	36 (68)	17 (32)		40 (75)	13 (25)		39 (74)	14 (26)		34 (64)	19 (36)		34 (64)	19 (36)	
SCC	36	29 (81)	7 (19)		27 (75)	9 (25)		23 (64)	13 (36)		28 (78)	8 (22)		31 (86)	5 (14)	

*NSCLC* non-small cell lung cancer, *AD* adenocarcinoma, *SCC* squamous cell carcinoma

### Correlations between SCP3 and VEGF family member expression

**Fig. 3:**
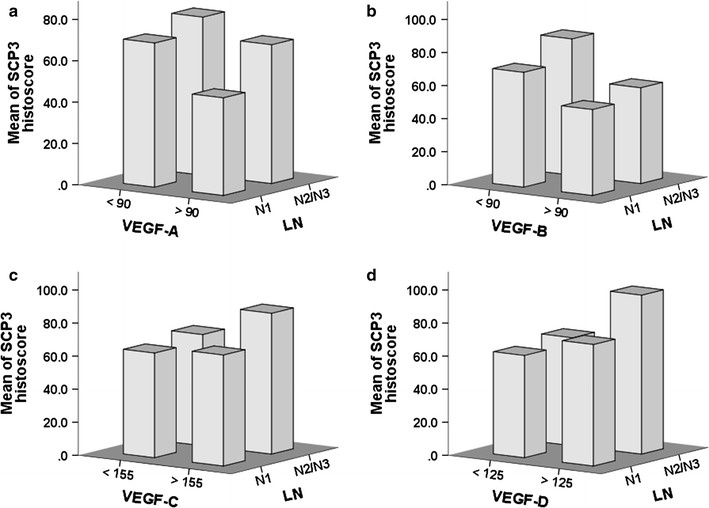
Association between SCP3 and VEGFs in human non-small cell lung cancer (NSCLC). Correlation between SCP3, N factor (pN1 and pN2–3), and VEGF-A (**a**), VEGF-B (**b**), VEGF-C (**c**) or VEGF-D (**d**)

To explore the association between SCP3 and lymphangiogenetic factors, we further examined correlation between SCP3 and VEGF family member expression using Pearson Chi square test (Table 2). SCP3 expression showed significant positive correlation with VEGF-C (odds ratio [OR] = 5.600, *P* < 0.001) and VEGF-D (OR = 7.700, *P* < 0.001), whereas SCP3 expression was negatively correlated with VEGF-A (OR = 0.205, *P* = 0.019) and VEGF-B (OR = 0.244, *P* = 0.019). Furthermore, SCP3 expression inversely correlated with increased VEGF-A and VEGF-B expression, and both VEGF-A and VEGF-B expressions levels also inversely correlated with LN metastasis status (Fig. 3).

**Table 2:** Association between SCP3 and VEGF-A, VEGF-B, VEGF-C or VEGF-D expression in NSCLC patients with LN metastasis

SCP3 expression				
	High no. (%)	Low no. (%)	Odds ratio (95% CI)	*p* value
VEGF-A			0.205 (0.044–0.955)	0.029
High	2 (9)	20 (91)		
Low	22 (33)	45 (67)		
VEGF-B			0.244 (0.066–0.905)	0.026
High	3 (11)	24 (89)		
Low	21 (34)	41 (66)		
VEGF-C			5.600 (2.032–15.435)	<0.001
High	14 (52)	13 (48)		
Low	10 (16)	52 (84)		
VEGF-D			7.700 (2.682–22.109)	<0.001
High	14 (58)	10 (42)		
Low	10 (15)	55 (85)		

*NSCLC* non-small cell lung cancer, *VEGF* vascular endothelial growth factor, *LN* lymph node, *CI* confidence interval

### Prognostic implications of SCP3 expression in lung cancer

**Fig. 4:**
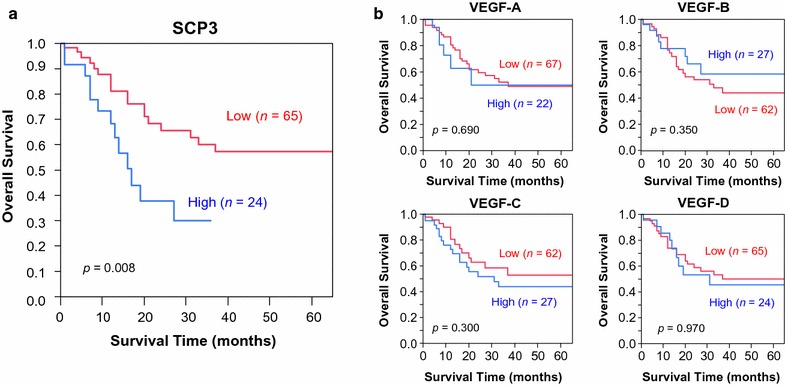
Kaplan–Meier survival curves for non-small cell lung cancer (NSCLC) patients with lymph node metastasis. **a** Patients with high SCP3 expression (median survival, 16 months) showed significantly worse survival than those with low SCP3 expression (median survival, 66 months). **b** No significance in survival differences was observed for patients with expression of different VEGFs

Analysis of patient survival was performed by Kaplan–Meier plots and log-rank analysis. Median survival time for patients with high SCP3 expression was significantly worse (median, 16 months) than that for patients with low expression (median, 66 months; *p* = 0.008; Fig. 4). No significant survival differences were observed for any tested VEGF family member. After adjustments for age, gender, cancer type, T factor and other antibodies, SCP3 remained significant (*P* = 0.008) and showed higher hazard ratio (HR) than T factor (HR; 1.86 with SCP3 and 1.80 with T factor) by a Cox proportional hazards regression model (Table 3).

**Table 3:** Multivariate analysis of the association between prognostic variables and overall survival in NSCLC

Variables	Hazard ratio (95% CI)	*p* value
Age	0.76 (0.47–1.27)	0.280
Gender	0.54 (0.30–0.92)	0.030
T factor	1.80 (1.04–3.43)	0.040
Cancer type	0.98 (0.66–1.50)	0.800
SCP3 expression	1.86 (1.17–2.91)	0.008
VEGF-A expression	1.05 (0.63–1.62)	0.840
VEGF-B expression	1.05 (0.67–1.50)	0.800
VEGF-C expression	1.20 (0.83–1.73)	0.330
VEGF-D expression	0.95 (0.58–1.51)	0.820

*NSCLC* non-small cell lung cancer, *CI* confidence interval, *VEGF* vascular endothelial growth

## Discussion

Lung cancer has poor prognosis because most patients present with advanced or metastatic disease at the time of diagnosis. The metastatic spread of malignant cells occurs via lymphatic or vascular spread and is considered to be one of the most critical prognostic factors in NSCLC. Indeed, the detection of cancer cells in lymphatic vessels and LNs is a key criterion in the staging of NSCLC and has been used to develop therapeutic strategies. Currently, the axis of VEGF-C/VEGF-D/VEGFR-3 thought to be a major mediator of tumor lymphangiogenesis. VEGF-C and VEGF-D overexpression in the mouse tumor model can induce lymphangiogenesis and promote lymphatic metastasis of tumor cells [21], whereas suppression of VEGFR-3 signaling inhibits LN metastasis in gastric cancer [22]. Although several studies have shown that the VEGF-C/VEGF-D/VEGFR-3 axis promotes lymphangiogenesis in cancer, the molecular mechanism of lymphangiogenesis is not yet fully understood.

Prior studies have shown VEGF-C and VEGF-D overexpression in tumor cells can led to the formation new lymphatic vessels and the promotion of LN and distant-organ metastases [19, 20, 23–27]. Overexpression of VEGF-C can certainly enhance tumor lymphangiogenesis as well as nodal and distant-organ metastasis [25, 26], whereas knockdown of VEGF-C can reduce LN and lung metastases [28, 29]. Wen et al. have also demonstrated that up-regulated VEGF-D expression can promote tumor-associated lymph angiogenesis and lymphatic metastasis using murine LN metastasis models [30, 31]. In prior study, we have shown that SCP3 overexpression was associated with T factor in the early stage of NSCLC patients [13]. Recent study have showed that SCP3 overexpression is associated with poor prognosis of patients with cervical cancer [12]. However, the association of SCP3 with VEGF-C, VEGF-D and lymphangiogenesis is unknown. In the present study, we demonstrate that SCP3 overexpression correlated with VEGF-C and VEGF-D expression in NSCLC patients with LN metastasis. We evaluated 68 patients without lymph node metastasis from the same patient population, and failed to find a relationship of SCP3 expression with VEGF-C or VEGF-D, nor was there an association with survival for any of the factors examined (data not shown). These data suggest that SCP3 is closely associated with some process in lymphangiogenesis in NSCLC. To the best of our knowledge, this is the first study to identify a positive correlation between SCP3 and VEGF-C or VEGF-D expression levels in NSCLC patients with lymph node metastasis.

We previously demonstrated that SCP3 was linked with LN metastasis of cervical cancer [12], whereas SCP3 was associated with poor outcome in early stage of NSCLC by immunohistochemistry (IHC) and manual visual scoring [13]. Although immunohistochemistry is providing excellent localization on the examined tissue, lacks quantification without sophisticated instrumentation and normalization tool in chromogenic applications. In previous study, we evaluated SCP3 expressional level by traditional visual scoring which is fraught with data quality problems. IHC data resulted from traditional manual scoring has good to excellent intra- and inter-observer reproducibility [32–34]. However, estimation of negative and positive percentages of areas stained has only poor to good reproducibility [16]. Notably, prior studies demonstrated that continuous IHC score data resulted from digital image analysis may allow identification of IHC cut-off points of prognostic relevance that are either undetected [35] or are less statistically significant [36, 37] by manual visual scoring. In the present study, we observed higher SCP3 positivity compared to the previous study [13]. This discrepancy in the findings may be resulted from employed IHC scoring method and lack of well-defined SCP3 cut-off values for positive and negative results. Although image analysis allows reproducible data with high throughput mode in the hands of a well-trained pathologist, a well-defined algorithm and cut-off values for SCP3 stain remains to be developed for the clinical utility.

The prognostic value of VEGF-A, VEGF-B, VEGF-C and VEGF-D is still controversial. A number of studies has shown a direct correlation between expression of VEGF-C or VEGF-D in tumor cells and the metastatic tumor spread of many human cancers [32–40]. Chen et al. reported that VEGF-C may be a predictor of early post-operative recurrence in patients with N2 NSCLC [35], and Maekawa et al. showed that VEGF-D expression indicated poor prognosis in lung cancer patients with T1 adenocarcinoma [39]. In contrast, Liao et al. showed VEGF expression was not associated with survival in NSCLC. Likewise, Tomita et al. reported that there is no relationship between VEGF expression and survival rate in patients with N2 NSCLC [41]. In this study, we observed that although VEGFs do not show prognostic significance, SCP3 expression is a prognostic factor. These discrepancies may be explained by the lack of standardized methodology, different standards of interpretation, or differences in patient population among the various studies.

To better understand the mechanism by which SCP3 promotes lymphangiogenesis, we examined SCP3, VEGF-C and VEGF-D expression in human lung cancer cell lines using western blotting. Overexpressed SCP3 positively correlated with VEGF-C and VEGF-D expression (Fig. 1b). Inhibition of SCP3 expression by SCP3 siRNA gene knockdown resulted in significant down-regulation of VEGF-C and VEGF-D expression. These results suggest that SCP3 may promote lymphangiogenesis by up-regulating VEGF-C and VEGF-D expression in tumor cells through an unknown signaling pathway. Among the known signaling pathways, the AKT pathway is a major candidate because it plays a pivotal role in transformation by inducing cell survival, proliferation, invasion, migration and angiogenesis [42, 43]. In prior study, we demonstrated that phospho-AKT levels were increased in SCP3-expressing cervical cell lines [11] and that SCP3 mediates an oncogenic phenotype of cervical cancer cells through an AKT-dependent pathway [12]. Further study is needed to demonstrate the details of SCP3’s involvement in lymphangiogenesis and whether there is any link between the up-regulation of lymphangiogenetic factors (VEGF-C and VEGF-D) and the AKT pathway.

## Conclusions

In conclusion, SCP3 is significantly associated with VEGF-C and VEGF-D expression and can be used to indicate poor prognosis in NSCLC with LN metastasis. It seems likely that SCP3 expression can influence lymphangiogenesis by VEGF-C and VEGF-D upregulation separately from angiogenesis. Thus, SCP3 may be a potential therapeutic target, considering its possibly pivotal role in lymphangiogenesis. Further study of SCP3’s molecular mechanism may lead to the development of a novel therapeutic target for NSCLC.

## Additional files

**Additional file 1: Figure S1.** The histoscore distribution of SCP3, VEGF-A, VEGF-B, VEGF-C, and VEGF-D expression by quantitative image analysis. The cut-off values were defined by consideration of the distribution and prognostic significance of the value.

**Additional file 2: Table S1.** Clinicopathological characteristics of patients (*n* = 89).

## Abbreviations

SCP3: synaptonemal complex protein 3
NSCLC: non-small cell lung cancer
LN: lymph node
VEGF: vascular endothelial growth factor
TNM: tumor-node-metastasis
siRNA: small interfering RNA
GFP: green fluorescent protein: TMA: tissue microarray
H&E: hematoxylin and eosin
OR: odds ratio
CI: confidence interval
