# Supplementary figures and images for: Synaptonemal complex protein 3 is associated with lymphangiogenesis in non-small cell lung cancer patients with lymph node metastasis
Source: J Transl Med. 2017 Jun 17;15:138. doi: 10.1186/s12967-017-1241-5 (PMC5473978; doi:10.1186/s12967-017-1241-5)

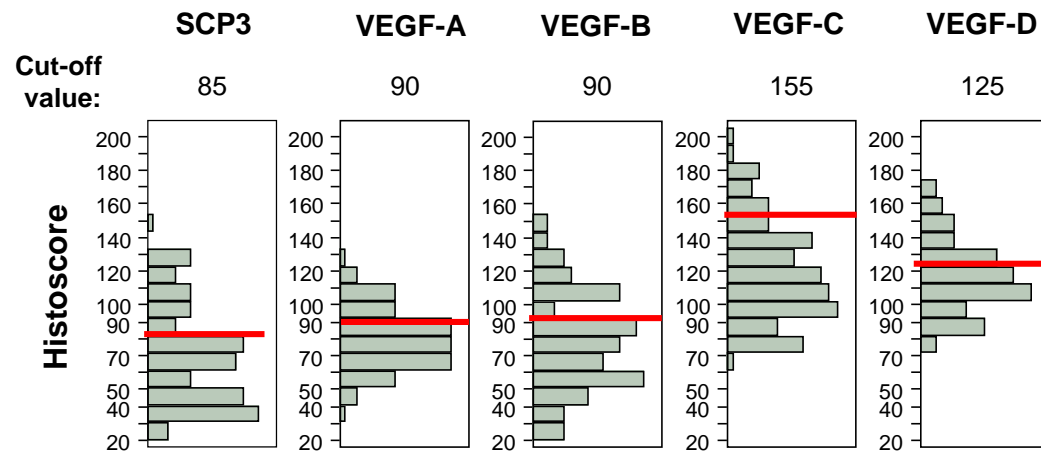

**Figure S1**

Supplement: Supplementary file 1 — Additional file 1: Figure S1. The histoscore distribution of SCP3, VEGF-A, VEGF-B, VEGF-C, and VEGF-D expression by quantitative image analysis. The cut-off values were defined by consideration of the distribution and prognostic significance of the value. [file 12967_2017_1241_MOESM1_ESM.pdf]
