# Supplementary material for: Synaptonemal complex protein 3 is associated with lymphangiogenesis in non-small cell lung cancer patients with lymph node metastasis
Source: J Transl Med. 2017 Jun 17;15:138. doi: 10.1186/s12967-017-1241-5 (PMC5473978; doi:10.1186/s12967-017-1241-5)
Supplement: Supplementary file 2 — Additional file 2: Table S1. Clinicopathological characteristics of patients (n = 89). [file 12967_2017_1241_MOESM2_ESM.pdf]

Supplementary table S1 Clinicopathological characteristics of patients (*n* = 89)

Variable	*Case No. (%)*
Gender	
Male	64 (71.9)
Female	25 (28.1)
Stage	
I	--
II	40 (44.9)
III	47 (52.8)
IV	2 (2.3)
T Stage	
T1	15 (16.9)
T2-4	74 (83.1)
N Status	
pN1	46 (51.7)
pN2-3	43 (48.3)
Tumor type	
Adenocarcinoma	53 (59.6)
Squamous cell carcinoma	36 (40.4)
